# How genomics can be used to understand host susceptibility to enteric infection, aiding in the development of vaccines and immunotherapeutic interventions

**DOI:** 10.1016/j.vaccine.2019.01.016

**Published:** 2019-08-07

**Authors:** Lynda Mottram, Subhra Chakraborty, Eric Cox, James Fleckenstein

**Affiliations:** a Department of Microbiology and Immunology, Institute of Biomedicine, Sahlgrenska Academy at the University of Gothenburg, Gothenburg, Sweden; b Department of International Health, Johns Hopkins Bloomberg School of Public Health, Baltimore, MD, United States; c Department of Virology, Parasitology and Immunology, Faculty of Veterinary Medicine, Ghent University, Merelbeke, Belgium; d Department of Medicine, Division of Infectious Diseases, Washington University School of Medicine, St. Louis, MO, United States; e Medicine Service, Veterans Affairs Medical Center, St. Louis, MO, United States

**Keywords:** Genomics, *Shigella*, ETEC, Host genetic factors, Vaccine antigen candidates, Host-pathogen interactions

## Abstract

Thanks to the modern sequencing era, the extent to which infectious disease imposes selective pressures on the worldwide human population is being revealed. This is aiding our understanding of the underlying immunological and host mechanistic defenses against these pathogens, as well as potentially assisting in the development of vaccines and therapeutics to control them. As a consequence, the workshop “How genomics can be used to understand host susceptibility to enteric infection, aiding in the development of vaccines and immunotherapeutic interventions” at the VASE 2018 meeting, aimed to discuss how genomics and related tools could be used to assist *Shigella* and ETEC vaccine development. The workshop featured four short presentations which highlighted how genomic applications can be used to assist in the identification of genetic patterns related to the virulence of disease, or host genetic factors that could contribute to immunity or successful vaccine responses. Following the presentations, there was an open debate with workshop attendees to discuss the best ways to utilise such genomic studies, to improve or accelerate the process of both *Shigella* and ETEC vaccine development. The workshop concluded by making specific recommendations on how genomic research methods could be strengthened and harmonised within the ETEC and *Shigella* research communities.

## 1 Introduction

Enterotoxigenic *Escherichia coli* (ETEC) and *Shigella* remain leading bacterial causes of diarrhoeal and dysenteric illness in children of underdeveloped countries, and in individuals who travel to such endemic areas [1]. There are currently no licenced vaccines against these enteric pathogens, but the global health community has prioritized their development [1], [2]. One way that could improve or accelerate the process of both *Shigella* and ETEC vaccine development might be to complement classical vaccinology approaches with the use of genomics and/or related genomic tools.

Genomic studies using both computational and experimental methods, have significantly advanced our understanding of the molecular pathogenesis of many microbes. For instance, whole genome sequencing (WGS)/next-generation sequencing (NGS) can be used to provide an insight into genomic diversity and evolution of different microbial species [3]. Reverse vaccinology methods can also be used to aid the identification of novel vaccine antigen candidates against different pathogens [4], [5]. Likewise, host gene expression profiling of disease burdened patients or vaccinees compared to healthy controls, has enabled the identification of immunologically related host biomarkers. This systems vaccinology approach can be used to inform vaccine efficacy, and the safety of novel vaccine candidates or immunotherapeutic interventions [6], [7], [8].

When considering the use of genomics, related genomic technologies and methods should also be considered. Examples include: transcriptomic analysis of host-pathogen interactions upon infection or vaccination; proteomic analysis to study protein-protein interactions within the pathogen or between the host and pathogen; pathogen and host rapid phenotyping; immunoproteomic analysis; and data mining of various genomic or protein databases [7], [8], [9], [10]. Such genomic tools are attempting to understand the similarities and differences between host immune responses to natural infection or vaccination, with the ultimate aim of defining better correlates of protection.

The purpose of this VASE 2018 workshop was to discuss how genomics and related applications could be applied to facilitate the development of *Shigella* and ETEC vaccines. The workshop began with four short presentations that highlighted how genomics and related tools can successfully be used to aid the identification of genetic patterns related to the virulence of disease, or host genetic factors that could contribute to immunity or successful vaccine responses. The presentations were then followed by an open workshop debate on the best ways to utilise such genomic based studies to improve ETEC and *Shigella* vaccine development. The workshop concluded by making specific recommendations on how genomic research methods could be strengthened and harmonised in the ETEC and *Shigella* vaccine development fields.

## 2 Summary of the presentations

### 2.1 *FUT2*, an association with ETEC infection

Lynda Mottram (University of Gothenburg, Sweden) described how she has used Genome-wide Association study (GWAS) data to identify potential human genetic biomarkers of severe ETEC infection susceptibility/possible ETEC vaccine efficiency [19].

There is evidence to suggest that the human small intestinal glycan antigen Lewis a (Le^a^), could be a human intestinal binding receptor of ETEC colonization factor I (CFA/I), and related colonisation factor (CF) fimbriae [11], [12]. A clinical trial has also previously demonstrated Le^a^ phenotyped Bangladeshi children are more susceptible to symptomatic than asymptomatic ETEC CFA/I infection [13]. The human Le^a^ phenotype (caused by homozygous *fucosyltransferase 2* [*FUT2*] single nucleotide polymorphisms [SNPs]) has also been previously associated with susceptibility to other enteric infections, as well as lower immunoglobulin A (IgA) antibody responses to rotavirus vaccination [14], [15]. Subsequently the aim of Dr Mottram’s study was to determine if a *FUT2* SNP could also be used as a human genetic biomarker of susceptibility to severe ETEC CFA/I and related CFs diarrhoeal disease/and or vaccine efficiency associated with ETEC vaccines containing CFA/I.

Dr Mottram initially searched for the frequency of known *FUT2* non-synonymous SNPs in the Bangladeshi population. This was performed using The 1000 Genomes Project dataset, a large GWAS open access catalogue of genetic variants (allele frequency [AF] > 0.01) found in 2,504 human genetic sequences of 26 different human populations worldwide [16]. Included in this dataset are 86 genetic sequences of healthy adults who live in Dhaka, Bangladesh.

Consequently, a variant calling file (VCF) from The 1000 Genomes Project server that contained all the *FUT2* genetic mutations (in region *FUT2*, chromosome 19: Genome Reference Consortium Human Build 37: 49199228:49209207) identified in the GWAS studied individuals, was downloaded [16]. The Ensembl allelic AF calculator was used to predict the total allele count and alternative allele count of all genetic variants (AF > 0,01) present in each of the 26 worldwide populations [17]. Then, to identify non-synonymous *FUT2* SNPs, a further analysis of The 1000 Genomes Project *FUT2* genetic variation dataset was performed using the Ensembl Variation Effect Predictor (VEP) tool [18]. This analysis of The 1000 Genomes project dataset identified three non-synonymous *FUT2* single nucleotide SNPs candidates, that were present in the Bangladeshi population [19].

Using RT-PCR SNP genotyping methods, the frequency of these three *FUT2* non-synonymous SNPs in Le^a^ phenotyped Bangladeshi children, who had been previously clinically monitored for ETEC infection during the first two years of life were then assessed [13], [19], [20]. This retrospective association study identified two *FUT2* SNPs; rs200157007-TT and rs601338-AA, that were strongly associated with symptomatic ETEC infection and the Le^a^ phenotype in the Bangladeshi children [19].

However, potentially due to the limited number of samples from which human gDNA could be successfully extracted from, only a trend but not a statistical relationship with rs200157007-TT and rs601338-AA SNPs, symptomatic ETEC expressing CFA/I or related CF infection, and the Le^a^ phenotyped Bangladeshi children could be found. Subsequently, further clinical studies in other ETEC endemic areas, as well as further *in-vivo* analysis are needed to evaluate if these *FUT2* SNPs could be used as host biomarkers of ETEC CFA/I (and related CFA/I CFs) infection susceptibility, or vaccine efficiency of ETEC vaccine candidates containing the CFA/I antigen.

### 2.2 Secreted ETEC virulence proteins drive pathogen-host interactions and contribute to clinical outcome

James Fleckenstein (Washington University School of Medicine, St. Louis, Missouri) explained how his team is complementing their host-pathogen interaction studies with genomics and related “omics” tools, to identify and characterise noncanonical ETEC vaccine candidates that are associated ETEC pathogenesis; i.e. novel ETEC virulence factors associated with ETEC adhesion, intestinal colonisation and toxin delivery during severe ETEC infection.

Initially, Dr Fleckenstein’s group performed a Tn*phoA* transposon-based mutagenesis study using the fully sequenced human challenge ETEC H10407 strain, to identify secreted or surface-expressed antigens. This study enabled the identification of a number of novel ETEC plasmid-encoded virulence loci, including the *eatA* autotransporter and the *etpBAC* two-partner secretion system, that were expressed in the virulent ETEC H10407 strain, but not in the fully sequenced *E. coli* K-12 strain [21], [22]. Consequently, EatA and EtpA are now being characterised to define their suitability as ETEC vaccine components, as well as their contribution to disease in naturally infected hosts.

By combining genomic database mining and immunoproteomics, EatA has been found to be a member of the serine protease autotransporter family, as well as being fairly conserved among a geographically and phylogenetically diverse group of ETEC strains [23]. In addition, EatA has been found to be highly immunogenic [24]. It has also been shown that EatA shares a high degree of homology with SepA, a virulence protein secreted by *Shigella flexneri* [21]. Moreover EatA has been found to degrade human MUC2, a major intestinal mucin, expressed by goblet cells of the human small intestine and colon [25]. Using an *in-vitro* enteroid (human small intestinal derived stem cells) model [26], it has also been observed that MUC2 degradation by EatA significantly enhances ETEC LT or ST toxin delivery to host intestinal cell surface receptors.

Likewise, EtpA has found to be a large glycosylated exoprotein, secreted via the etpBAC two-partner secretion system (TPS) [22], and acts as an adhesin molecule, forming a molecular bridge between the tips of ETEC flagella and the host epithelial surface [27]. Furthermore and similar to EatA, genomics and molecular studies have demonstrated that EtpA appears to be conserved among a diverse group of ETEC strains [23], as well as being highly immunogenic [24].

Using a combination of human glycan arrays, biolayer inferometry, noncanonical amino acid labelling and hemagglutination studies, EtpA has been identified as a dominant ETEC blood group A–specific lectin/hemagglutinin [28]. To demonstrate further that this EtpA-blood group A mediated binding interaction enhances ETEC pathogenesis, Dr Fleckenstein’s group has also used the blood group A-expressing HT-29^+/+^ wild-type intestinal cell line and blood group A knockout HT-29A^−/−^ cells (derived by CRISPR-Cas9 engineering to eliminate the gene encoding blood group A glycotransferase) [29], as well as enteroids from blood group A individuals, to demonstrate that EtpA-blood group A mediated interaction significantly enhances bacterial adhesion ETEC LT and ST toxin delivery [28].

Such molecular and genomic characterisation of EtpA has enabled further collaborations with Dr Chakraborty (see Section 2.3) to show that diarrhoeal illness following ETEC H10407 controlled human infection model (CHIM) challenge tends to be significantly more severe in human blood group A volunteers than volunteers with other blood group phenotypes [28], [30].

### 2.3 Impact of host factors in preclinical diarrhoea outcome after infection with ETEC in humans

In the third presentation, Subhra Chakraborty (Department of International Health of the John Hopkins Bloomberg School of Public health, Baltimore, MD) described how genomics is being used to evaluate human host responses to ETEC during CHIM studies.

A total of 30 naive ETEC H10407 subjects were enrolled into a CHIM study, in an inpatient unit at the Centre for Immunization Research, John Hopkins University. These volunteers were randomly assigned to one of two dosing groups; 10^5^ or 10^6^ colony-forming unit (CFU) of ETEC strain H10407 (LT+ ST+ CFA/I+ and O78+). Following the ETEC H10407 challenge, 29 out of the 30 volunteers were found to shed ETEC bacteria. However, the rates of moderate to severe diarrhoea (MSD) compared to asymptomatic ETEC carriage varied between the volunteers of the same dosing cohorts [30]. Subsequently, a comparison was made to compare the results of MSD patients to asymptomatic ETEC shedders to identify potential host biomarkers of severe diarrhoeal ETEC illness.

Dr Chakraborty pre-screened the volunteers for pre-existing ETEC antibody titres before ETEC H10407 challenge. This was performed using antibodies in lymphocyte supernatants (ALS), sera and faecal ELISA assays, and included; lipopolysaccharide (LPS) IgA and IgG, LTB IgA and IgG, and CFA/I IgA and IgG analysis. Dr Chakraborty compared subjects who subsequently developed asymptomatic (n = 24) verses MSD (n = 6) subjects, and observed only significantly higher pre-challenge LTB IgG antibody sera titres (*P =* 0.02) in the asymptomatic subjects [31].

Next, Dr Chakraborty described the analysis that compared the whole peripheral blood RNA expression profiles of MSD (n = 6) versus asymptomatic (n = 6) at the baseline time point [31]. DNA Microarray (using the Affymetrix GeneChip Microarray Human Genome U133A2.0) analysis was used to identify genes associate with susceptibility to ETEC disease in these volunteers challenged with ETEC H10407.

This microarray analysis identified 29 differentially expressed gene probes that were potentially associated with resilience to severe ETEC infection. Of these 29 identified probes, gene probe sets associated with major histocompatibility complex (MHC) protein binding and MHC class I protein binding molecules were significantly up-regulated in the asymptomatic dataset. The analysis also identified four tubulin genes (*Tubb2A*, *Tubb2b*, *Tubb3* and *Tubb4B*) which have previously been associated with *E. coli* pathogenesis, to be up-regulated in the asymptomatic (resilient to infection) dataset. In comparison, *C4BPA* an inhibitor gene associated with the classical complement pathway was down-regulated in the asymptomatic (resilient to infection) dataset [31].

Dr. Chakraborty also described the 16S rRNA (ribosomal RNA) gene sequencing gut microbiome analysis performed on stool specimens from a subset of these ETEC H10407 infected individuals; MSD (n = 5) and asymptomatic (n = 6) individuals. The aim of this analysis was to evaluate if pre-infection microbiota could be used to predict the onset of severe diarrheal ETEC disease [32].

Results from the 16S rRNA analysis revealed the MSD individuals (i.e. potential pre-infection microbiota predictors associated with severe ETEC disease) had a higher concentration of faecal *Escherichia* as well as *Bacteroides dorei*, *Prevotella* species, *Alistipes onderdonkii*, *Bacteroides* species (*ovatus*), and *Blautia* species. In contrast, the faecal microbiota of the asymptomatic carriers (i.e. potential pre-infection microbiota predictors associated with resistance against ETEC diarrhoeal disease) were enriched with normalised 16S rRNA gene sequences including *Sutterella* species, *Prevotella copri*, and *Bacteroides vulgatus* [32].

### 2.4 Genetic susceptibility of pigs to infections with enterotoxigenic and shiga toxin producing *E. coli*

In the final presentation, Eric Cox (Ghent University, Belgium) described the use of genomics and related genomic tools to examine the genetic susceptibility of pigs to F18+ fimbriae ETEC/shigella toxin-producing *E. coli* (STEC) and F4+ fimbriae ETEC strains. The susceptibility of pigs to F18+ and F4+ *E. coli* is determined by the presence of F18 and F4 specific host receptors in the brush boarder of pig’s small intestine. Therefore, understanding the genetic composition of these F18 and F4 host receptors is useful for animal health and subsequent breeding management.

The functional pig F18+ *E. coli* intestinal binding receptor had been previously thought to be only encoded by *FUT1*, a host gene that encodes for α(1,2) fucosyltransferase blood group AO antigens on small intestinal type 2 glycan chains in pigs. Meijerink et al. [33] had demonstrated that a *FUT1* SNP at nucleotide position bp307 (G=>A transition) of the *FUT1* open reading farm (ORF) was closely linked to susceptibility to F18+ *E. coli* infection, and thus genetic variations in this *FUT1* M307 SNP can be used as a specific genetic marker for selecting and breeding pigs which are resistant to F18+ *E. coli* infections [33], [34].

Conversely, Prof. Cox’s group has discovered that F18+ fimbriae attach to the small intestine of young piglets by binding to mucosal type 1 core glycans chains that express A/O blood group determinants, with this host-pathogen interaction directly correlating with F18+ *E. coli* infection susceptibility [35]. Subsequently, using structural and site directed mutagenesis studies it was defined that FedF, the N-terminal domain subunit of F18 fimbriae is responsible for F18+ *E. coli* binding to pig A and O blood groups [36], [37].

Recent genetic studies have further identified that piglet susceptibility to F18+ *E. coli* infection might not be an absolute correlate with the *FUT1* M307 related SNP. Prof. Cox’s group is currently analysing data to suggest the genetic regulation of *FUT2* (a gene closely related to *FUT1*), might also be controlling blood group A and O blood expression in the pigs small intestine, and thus piglet susceptibility to F18+ *E. coli* infection. Interestingly, the expression of AO antigens in the pig’s small intestine is also directly correlate with age, as Prof. Cox’s group has noticed that new-borns are always resistant to F18+ *E. coli* infection independent of the *FUT1* M307 SNP, and that susceptibility to F18+ *E. coli* infection becomes highest just after weaning at 6–8 weeks old [38]. The mechanism regulating this age-related expression is not known.

Prof. Cox also described the current work to genetically define the F4+ ETEC fimbriae binding receptor in the porcine small intestine. There are three antigenic variants of F4+ ETEC fimbriae (F4ab+, F4ac+, F4ad+), with each antigenic variant showing a different binding pattern to brush border membrane proteins of small intestinal enterocytes. Only a small number of piglets completely lack an intestinal receptor to F4+ ETEC fimbriae, and are therefore resistant to F4+ bacteria and subsequent diarrhoea caused by F4+ ETEC strains. In Belgium however, most Flemish farmed pigs express F4ab or F4ac intestinal receptors, so Flemish pigs are subsequently more susceptible to F4ab+ or F4ac+ associated ETEC infections [39].

Several studies located the F4ab/acR locus(loci) on chromosome 13 and several linkage studies proposed different loci as candidate receptor loci such as the transferrin, the transferrin receptor (*TRFC*), mucin 4 (*MUC4*), mucin 13 (*MUC1*3), mucin 20 (*MUC20*), solute carrier family 12 member 8 (*SLC12A8)*, myosin light chain kinase (*MYLK*), karyopherin alpha 1 (*KPAN1*), beclin-1 associated RUN domain containing protein (*KIAA0226*), lactosylceramide 1,3-N-acetyl-β-D-glucosaminyl transferase (*B3GNT5*) loci, but none of the polymorphisms were found to be causative. Therefore, the Gent University researchers conducted a GWAS study using 120 precisely F4ab/ac receptor phenotyped Flemish pigs (i.e. 52 F4ab+/Fac+ *E. coli* resistant and 68 F4ab+/Fac+ *E. coli* susceptible pigs) from 5 different Belgium farms, based on *MUC4* and *MUC13* polymorphism and *in vitro* villous adhesion of F4 = and F4ac+ *E. coli*. DNA was isolated from the blood samples of these phenotyped pigs and then sequenced using the Porcine SNP60 BeadChip (Illumina) microarray [39], [40].

This GWAS study revealed that pig F4ab/ac ETEC susceptibility, is instead likely highly associated with two SNPs and genetic regions adjacent to *MUC13* (chr13: 144,810,100–144,993,222). Unfortunately, these genetic regions lacks annotated genes, and contain a sequence gap based on the sequence of the porcine GenomeBuild 10.2. Subsequently, it can only be currently hypothesise that a porcine orphan gene or trans-acting element in the candidate region determines F4ab/F4ac ETEC susceptibility in pigs [40]. Prof. Cox therefore proposes further genetic and functional annotation studies to identify the exact mechanisms and porcine host receptor structures of F4ab/ac ETEC fimbriae.

## 3 Summary of the group discussion

Following the presentations, the workshop moved to an open discussion with attendees on how genomic and related genomic tools could be best utilised to accelerate the current process of ETEC and *Shigella* vaccine development.

To potentially aid the development of new vaccines, a good part of the workshop discussion centred on the use of genomics during molecular epidemiology studies. The assuming advantages of this is that WGS or related high throughput genomic platforms could be used to characterise the genomic diversity of the pathogen, as well as take into account the genetic/immunological variability’s of the host. Such information could be used to critically define host-pathogen dynamics during infection, and thus define correlates of protection.

For this to happen, some workshop attendees suggested such studies would have to be large-scale multi country epidemiological studies (e.g. similar to the scale of the GEMS study), where strong population data and phenotypic information was collected on both the pathogen and the host. To aid the subsequent identification of any genomic host biomarker, associated with infection susceptibility, these epidemiological studies would also importantly need to record the immune response elicited in each infected individual. Another alternative option to using large-scale epidemiological studies could be to use CHIM studies to define host-pathogen dynamics during infection in a smaller number of individuals, in a controlled environment [41]. Subsequent findings could then be longitudinally evaluated in further field studies.

However, the workshop discussion emphasized that for genomics analysis to be used efficiently in such molecular epidemiological or CHIM studies, it would be important for both the ETEC and *Shigella* scientific communities to harmonise which infection time points are used to collect data related to immunological and genomic analysis. Furthermore, to ensure that sample collection does not affect the related genomics results obtained, it might also be important to standardise what human samples are collected, and how they are collected and stored at both the epidemiological field site or in the clinic.

Likewise, other workshop attendees suggested that WGS/NGS can be used to fully annotate a group of ETEC or *Shigella* reference strains that represent their worldwide geographical, temporal and phenotypic diversity. It is well recognised the role played by the erstwhile WHO reference laboratories, NIH, CDC, USDA as well those such as the Sanger Institute in generating genomic databases of *Shigella* and ETEC stains, who make reference strains/genomic sequences available to the scientific community. However, perhaps it could be advantageous to the ETEC and *Shigella* scientific communities to work with a harmonised set of genomically annotated ETEC and *Shigella* reference strains, as well as their related genomic sequences. Such a harmonised, defined and fully sequenced strain collection could be used in all the associated ETEC and *Shigella* vaccine developmental studies performed by different laboratories worldwide.

The workshop speaker’s sessions highlighted how high throughput genomic screening for virulence factors could offer potential for the rational development of new vaccine candidates, which could stand-alone or complement the vaccines currently in development. Workshop attendees highlighted use of proteomics [24] and the data mining of genomic or protein databases [9], to aid the identification and characterisation of vaccine genes or protein antigen candidates that play a key role in a pathogens ability to infect and the hosts immune response. Equally, the reverse vaccinology approach where comparative *in-silico* analysis of multiple whole genome sequences are used to identify highly conserved antigen in pathogenic strains but not commensal strains was also mentioned as a successful method of vaccine discovery[5].

Similarly, the group discussed the advantages of genomic analysis during preclinical *in-vivo* vaccine developmental studies. Animal studies are considered to be very important to evaluate vaccine efficacy. However, some animal models never perfectly correlate with the response in humans to ETEC or *Shigella* infection. Subsequently, as well as using gene edited bacterial strains to characterise vaccine antigen candidate expression and function, it was discussed that genomics analysis could also be used to define specific host-pathogen interactions during infection. Perhaps also, genomics analysis could also be used in animal studies to assess for the presence of a specific genetic biomarker that predict the mucosal efficiency or toxicity of a vaccine, or even how effective a vaccine would be in humans.

Others suggested the use of high throughput screens using large scale sero-epidemiological studies [42] or gene-engineered knockout (e.g. using CRISPR/Cas9 and targeted genome engineering) mammalian cells lines or mice, to identify host biomarkers genes associated altered susceptibility to specific pathogenic antigens [43], [44], [45], [46]. In addition, genomics could be used to genetically define small intestinal organoid or enteroid models to eliminate translation of results from animals to human models [26], [47].

Following epidemiological studies, searches for antigens, *in-vitro* and *in-vivo* modelling, a vaccine next goes into clinical trial. Here the group discussed if genomics could also be used to improve the process by identifying and testing for biomarkers that alert researcher to toxicity or efficiency issues early in the clinical trial process. Moreover, using GWAS studies, it may also possible to identify the most genetically susceptible populations for a particular disease and thereby reduce the sample size needed for an effective trial, which could also reduce the cost of such clinical trials. However, whilst the group felt this was important, it was considered that further research is still necessary to identify such defined host biomarker(s) in the ETEC and *Shigella* fields.

## 4 Key recommendations

The workshop emphasised the importance of scientific interaction among members of the ETEC and *Shigella* research communities, and the harmonisation and standardisation of efforts to facilitate exchange of genomic sequencing, information and materials. The specific recommendations proposed from this workshop are:

1. Genomics and related genomic applications can be used to complement classical vaccinology approaches
2. To effectively use genomics and related technologies in ETEC and Shigella vaccine development, immunological and genomic sample collection time points should be standardised and harmonized amongst different laboratories
3. The types of sample (i.e. saliva blood, faeces, PBMCs) collected from the host for genomic related analysis should be standardised, to avoid genomic skewing of data
4. To standardise genomic testing in the ETEC and *Shigella* scientific communities, genomic collection and storage standard operating procedures (SOPs) could be established for use amongst the different ETEC and *Shigella* research groups
5. Establish a harmonised global set of genomically annotated ETEC and *Shigella* reference strains for use in CHIM, vaccine antigen discovery, and host-pathogen interaction studies
6. Use genomics to aid the development of more genetically defined humanised models for use in ETEC and *Shigella* pathogenesis studies.
